# Functional and molecular evidence for heteromeric association of P2Y_1_ receptor with P2Y_2_ and P2Y_4_ receptors in mouse granulocytes

**DOI:** 10.1186/s40360-016-0072-y

**Published:** 2016-07-07

**Authors:** Antonio Carlos Ribeiro-Filho, Marcus Vinicius Buri, Carlos Castilho Barros, Juliana Luporini Dreyfuss, Helena Bonciani Nader, Giselle Zenker Justo, Rogério Bastos Craveiro, João Bosco Pesquero, Antonio Miranda, Alice Teixeira Ferreira, Edgar Julian Paredes-Gamero

**Affiliations:** Centro Interdisciplinar de Investigação Bioquímica, Universidade de Mogi das Cruzes, Av. Dr Cândido Xavier de Almeida Souza, 200, Mogi das Cruzes, São Paulo Brazil; Departamento de Bioquímica, Universidade Federal de São Paulo, Rua Pedro de Toledo, 669 - 9° andar - Prédio de Pesquisa II, R. Três de Maio 100, São Paulo, São Paulo Brazil; Departamento de Nutrição, Universidade Federal de Pelotas, R. Gomes Carneiro, n°1, 96010-610 Pelotas, Rio Grande do Sul Brazil; Departamento de Biofísica, Universidade Federal de São Paulo, R. Botucatu 862, São Paulo, São Paulo Brazil

**Keywords:** P2Y receptors, Calcium signaling, Heteromeric association, Granulocytes

## Abstract

**Background:**

All hematopoietic cells express P2 receptors, however pharmacological characteristics such as expression and affinity in granulocytes are unknown.

**Methods:**

Pharmacological characteristics of P2 receptors were evaluated by Ca^2+^ measurements using Fura-2 fluorophore. P2 receptors expression were analyzed by flow cytometry and RT-PCR. P2 interaction were shown by coimmunoprecipitation, western blotting and FRET.

**Results:**

Granulocytes were responsive to P2Y agonists, whereas P2X agonists were ineffective. Ca^2+^ increase, elicited by ADP and UTP was dependent on intracellular stocks and sensitive to G-coupled receptor inhibition. Moreover, MRS2179, a specific antagonist of the P2Y_1_ receptor, abolished ADP response. Interestingly, ADP and UTP exhibited full heterologous desensitization, suggesting that these agonists interact with the same receptor. The heteromeric association between P2Y_1_ receptor and the P2Y_2_ and P2Y_4_ receptors was shown by immunoprecipitation and FRET analysis.

**Conclusion:**

Clear evidence of heteromeric association of P2Y receptors was found during the evaluation of P2 receptors present in mice granulocytes, which could impact in the classical pharmacology of P2Y receptors in granulocytes.

## Background

Many G protein-coupled receptors, such as those in the P2 receptor family, form homo- or hetero-associations. P2 receptors, which are activated by ATP and analogs, are classified as metabotropic P2Y or ionotropic P2X receptors. Heterodimerization involving P2X receptors is well known and receptor heterodimers P2X_2–3_, P2X_2–6_, P2X_4–6_ and P2X_1–5_ have been previously described [1, 2]. Some studies have shown that some P2Y receptors such as P2Y_1_ receptors, can form heteromeric association with adenosine A_1_ receptor [3] and P2Y_11_ receptor [4]. Additionally, P2Y receptors can form constitutively functional dimers or oligomers and homo- or hetero- associations that can alter the pharmacology and intracellular signaling of P2Y receptors [5–7]. Among P2Y receptors, pyrimidine receptors seem to have appropriate domains for interaction with other P2Y receptors [7].

ATP is the main physiological agonist that activates all P2 receptors, except P2Y_14_ receptor [8]. Some P2Y receptors (e.g., P2Y_1_, P2Y_12_ and P2Y_13_ receptors) can be activated by purine diphosphates such as 2meSADP or ADP [9, 10]. Pyrimidines such as UTP activate P2Y_2_ and P2Y_4_ receptors and UDP activates P2Y_6_ receptors [8, 11]. P2Y_1_, P2Y_2_, P2Y_4_, P2Y_6_ and P2Y_11_ receptors are mainly G_q/11_-protein coupled receptors, whereas P2Y_12_, P2Y_13_ and P2Y_14_ receptors are mainly G_i_-protein-coupled receptors [1, 10]. The only known P2 receptor capable of triggering adenylate cyclase in response to ATPγS or ATP is the P2Y_11_ receptor [12]. On the other hand, the ionic-channel family of P2X_1–7_ receptors increases Na^+^ and Ca^2+^ concentrations by competition. These receptors are activated by ATP, αβmeATP, βγmeATP and BzATP, whereas ADP is only a partial agonist of P2X_5_ and P2X_6_ receptors [13]. For P2X_1–7_ receptors, UTP does not activate any of the seven P2X receptors.

In the present study, we observed that ADP and UTP exhibited full heterologous desensitization in mice granulocytes, suggesting that these agonists interact with the same receptor. This unique phenomenon could be possible due to the heteromeric association observed between P2Y_1_/P2Y_2_ and P2Y_1_/P2Y_4_. These associations could promote changes in the classical pharmacological properties of P2Y receptors observed in granulocytes.

## Methods

### Chemicals

Adenosine 5’-triphosphate (ATP), α,β-methyleneadenosine 5′-triphosphate (αβMeATP), 2′-3′-O-(4-benzoylbenzoyl)-adenosine 5’-triphosphate (BzATP), βγ-methyleneadenosine 5’-triphosphate (βγMeATP), adenosine 5′-diphosphate (ADP), pertussis toxin (PTX), digitonin, hydrocortisone, Hystopaque (1119 and 1077) solutions and 2′-deoxy-N6-methyl adenosine 3′,5′-diphosphate diammonium salt (MRS2179) were purchased from Sigma Chemical Co. (MO, USA). Pluronic acid, 1-[6-[((17β)-3-Methoxyestra-1,3,5 [10]-trien-17-yl)amino]hexyl]-1H-pyrrole-2,5-dione (U73122), ADP and two different uridine 5′-triphosphates (UTP) were purchased from Calbiochem (CA, USA). Trizol, agarose, amplification grade deoxyribonuclease I, RnaseOUT-Rnase inhibitor, TaqDNA polymerase, fura-2 acetoxymethyl ester (fura-2/AM), fluo-4/AM, Alexa Fluor 488-conjugated goat anti-rabbit IgG and Iscove’s modified Dulbecco’s medium (IMDM) were obtained from Invitrogen Life Technologies/Molecular Probes (CA, USA). FACs lysing solution and the monoclonal extracellular antibodies Phycoerythrin (PE)-conjugated rat anti-Mac-1 and Cy7/PE-conjugated rat anti-Gr-1 were purchased from PharMingen Becton Dickinson (CA, USA). The polyclonal rabbit anti-P2Y (P2Y_1_, P2Y_2_, P2Y_4_, P2Y_6_, P2Y_11_ and P2Y_12_) antibodies were purchased from Alomone Labs (Jerusalem, Israel). Protein-A Sepharose was purchased from GE Healthcare (USA). Horse serum was obtained from StemCell Technologies Inc. (Vancouver, BC, Canada). All other chemicals were acquired from Merck-Calbiochem (Rio de Janeiro, Brazil). PTX and MRS2179 were diluted in water. U73122 and fMLP were diluted in >0.001 % DMSO at final concentration, controls were carried out to exclude alterations induced by DMSO.

### Isolation of granulocytes

Bone marrow was obtained from the femur bones of 3-month-old female C57BL/10 mice killed by rapid cervical dislocation. Bone marrow cells were flushed out from two femurs with Tyrode’s solution (3 ml) with a fitted syringe. The experimental protocols for animal care and use were reviewed and approved by the Bioethics Committee of our Institution according to the “Guide for the care and use of laboratory animals” approved by the Animal Care Ethics Committee of the Federal University of São Paulo (1464/03).

Bone marrow cell types were separated by gradient centrifugation with different density solutions. A pool of bone marrow from six mice was suspended (6 ml) in Hystopaque (d = 1.119 g/cm^3^, 3 ml and d = 1.077 g/cm^3^, 3 ml) and centrifuged (700 × g, 30 min), allowing separation of the cell types into distinct layers.

After separation of cell types, contaminants in the granulocytic fraction were removed. Erythroid cells were lysed with identical volumes of cool hypotonic (0.2 %) and hypertonic (1.6 %) NaCl solutions. Mononuclear contaminants were eliminated by centrifugation (200 × g, 10 min, 3 cycles) [14].

### Calcium measurements

#### Measurements with calcium fluorophore fura-2 in cell suspension

Cytoplasmic Ca^2+^ concentration ([Ca^2+^]_cyt_) was measured in a loaded cell suspension (10^6^ cells/ml) in Tyrode’s solution (137 mM NaCl, 2.68 mM KCl, 1.36 mM CaCl_2_, 0.49 mM MgCl_2_, 12 mM NaHCO_3_, 0.36 mM NaH_2_PO_4_, and 5.5 mM D-glucose). Incubations with Ca^2+^ indicator were performed at room temperature with fura-2/AM (2 μM) and pluronic acid (0.02 %) under constant shaking for 40 min approximately [15]. After incorporation of fura-2, the cells were washed and suspended in Tyrode’s solution (2.5 ml) and transferred to a quartz cuvette for fluorometer measurements (SPEX FluoroLog-2, AR-CM System). Changes in [Ca^2+^]_cyt_ were measured at 37 °C with alternated excitations at λ_Ex_ 340 nm and 380 nm, and emission was measured at λ_Em_ = 505 nm. The maximum fluorescence ratio (R_max_) was determined after disruption of the cell plasma membranes with digitonin (50 μM), and a minimum fluorescence ratio (R_min_) was obtained with MnCl_2_ (2 mM), followed by addition of EGTA (10 mM) in alkaline medium. [Ca^2+^]_cyt_ was calculated using the equation derived by Grynkiewicz et al. [16].

### Measurements with calcium fluorophore fluo-4 in long-term bone marrow cultures

To create a stromal layer, femur bones were excised from mice (C57BL/6) and their medullar cavities were aseptically flushed with Iscove's Modified Dulbecco's Medium (IMDM). These cells were seeded on glass coverslips (25 mm). The flasks were incubated (37 °C, 5 % CO_2_). Half of the media in each flask was replaced weekly with equal amounts of fresh media. The IMDM medium was supplemented with 5 % bovine fetal serum, 20 % horse serum and 10^−6^ M hydrocortisone. At the end of the eighth week, after stroma formation, the remaining hematopoietic cells were removed. New bone marrow from additional mice was collected in IMDM supplemented medium (10 ml) and cultured (2 h) in tissue culture flasks (75 cm^2^). Non-adherent cells were collected by removing the medium and were added (10^6^ cells per well) to the pre-cultured stroma [17].

For [Ca^2+^]_cyt_ measurements, the cells were incubated (40 min, at room temperature) with fluo-4/AM (10 μM) and washed with Tyrode solution. Images were obtained in two Z planes with a microscope equipped with a laser scanner and an objective (Plan-Neofluor, 63x/1.43 numerical aperture) under oil immersion (Leica, SP8, Germany). The fluo-4 probe was excited at 488 nm and emission was detected by using a bypass filter (λ_Em_ = 500–550 nm). The pinhole device was not used for [Ca^2+^]_cyt_ measurements. Images were collected at 4.5 s intervals for about 2 min. Fluorescence intensity was normalized by reference to the basal fluorescence using Examiner 3.2 (Zeiss, Germany) and Spectralyzer (Philadelphia, USA) software [17].

### Flow cytometry analysis

To determine the level of P2 receptor expression in granulocytes, 10^6^ bone marrow cells were fixed with 2 % formaldehyde for 30 min and permeabilized for 15 min with 0.01 % saponin. The cells were incubated for 2 h with rabbit anti-P2Y IgG antibodies (6 μg/ml anti-P2Y_1_, 8 μg/ml anti-P2Y_2_, 3 μg/ml anti-P2Y_4_, 6 μg/ml anti-P2Y_6_, 6 μg/ml anti-P2Y_11_ and 8 μg/ml anti-P2Y_12_) in 1 % bovine serum albumin (BSA) dissolved in phosphate-buffered saline (PBS). After addition of the first label, the cells were incubated for 40 min with Alexa Fluor 488-conjugated goat anti-rabbit IgG antibody (4 μg/ml). The cells were then incubated for 20 min with rat Cy7/PE-conjugated anti-Gr-1 (2 μg/ml) and rat PE-conjugated anti-Mac-1 (0.1 μg/ml). Ten thousand Gr-1^+^Mac-1^+^ events were collected with a cytometer (FASCalibur, Becton Dickinson). Data analysis was performed using CellQuest software (Becton Dickinson).

### RNA extraction and reverse transcription polymerase chain reaction (RT-PCR)

RNA extraction and RT-PCR were performed as previously described by Paredes-Gamero et al. [18] using the same primers for P2Y_1_, P2Y_2_ and P2Y_12_ receptors. The following oligonucleotide primers were used for amplifying nucleotide sequences of P2Y_4_ receptors: sense primer, 5’agcccaagttctggagatggtg3’; anti-sense primer, 5’ggtggttccattggcattgg3’ (GeneBank accession no. NM020621.3). The PCR amplification of the nucleotide sequence of P2Y_4_ receptors was performed by incubating the samples at 94 °C for 1 min followed by 35 cycles of 94 °C for 1 min and 54 or 56 °C for 1 min, with a final incubation for 7 min at 72 °C. At the end of the amplification, the strands were dissociated. Electrophoresis was performed in 1 % agarose gel with a 100-bp DNA ladder as a size marker. The bands were visualized with ethidium-bromide staining.

### Coimmunoprecipitation and western blotting

The granulocyte plasma membranes were disrupted with a lysing buffer that contained a protease inhibitor cocktail (1 % Triton X-100, 10 % glycerol, 5 mM EDTA, 4 mM benzamidine, 5 mg/ml ε-aminocaproic acid, 10 mM iodoacetamide in 20 mM Tris buffer, pH 8.0). The protein cell lysate (600 μg) was cleared by incubation (4 °C, 1 h) with rabbit normal serum (1:50). Protein A-Sepharose was added and the unspecific immunoprecipitate was used as a negative control. Subsequently, the lysate was incubated (4 °C, overnight) with rabbit polyclonal anti-P2Y IgG (12 μg/ml anti-P2Y_1_, 16 μg/ml anti-P2Y_2_, or 6 μg/ml anti-P2Y_4_) on a rotator. The samples were then incubated (4 °C, 6 h) with protein A-Sepharose (50 ml) on a rotator, centrifuged (700 × g, 2 min) and washed (3 times) with lysing buffer. The immune-complex was eluted from the protein A-Sepharose resin by adding the sample buffer (100 ml) for SDS-PAGE under reduced conditions (40 % glycerol, 8 % SDS, 0.2 M Tris–HCl, pH 6.8 and 5 % β-mercaptoethanol). The samples were boiled (5 min) to release the immune-complex. Immunoprecipitated proteins were added to SDS-PAGE (3–20 % acrylamide) [19], and Western blot analysis was used to determine whether the P2Y_1_ receptor interacted with other P2Y receptors. The proteins were electrotransferred to a nitrocellulose membrane. After blocking with non-fat skimmed milk (5 % solution) in washing buffer (0.05 % Tween in PBS), the receptors immunoprecipitated in the blot were detected with rabbit anti-P2Y IgG (2 μg/ml anti-P2Y_1_, 4 μg/ml anti-P2Y_2_ or 1 μg/ml anti-P2Y_4_) antibodies. Subsequently, horseradish peroxidase-conjugated goat anti-rabbit IgG was used as a secondary antibody and the reactive bands were visible after addition of enhanced chemiluminescent substrates.

A second sample was lysed as described above. After centrifugation at 12,000 g for 15 min at 4 °C, the supernatant containing the whole protein lysate (WP) was saved. Protein A-Sepharose was incubated with goat polyclonal anti-P2Y_1_ (16 μg/ml) on a rotator. Further, part of the WP (1,000 μg) was added and incubated at 4 °C for 12 h on a rotator, centrifuged (700 × g, 2 min) and washed (3 times) with lysing buffer. The immune-complex was eluted from the protein A-Sepharose resin by adding 100 μL of the sample buffer [19] and boiled for 5 min. Immunoprecipitated proteins and WP lysate were added to 10 % SDS-PAGE. After transfer, the PVDF membrane was blocked with blocking buffer (Odyssey™ Blocking Buffer- Li-COR) and incubated with rabbit anti-P2Y_2_ IgG, washed with PBS-Twin, and incubated with IRDye 800CW Donkey anti-Rabbit IgG (in green; Li-COR). After stripping, the membrane was blocked again and incubated with rabbit anti-P2Y_1_ IgG followed by IRDye 680RD Donkey anti-Rabbit IgG (in red – LI-COR). The fluorescence was read using the Odyssey CLx® Infrared Imaging System (Li-COR).

### Förster resonance energy transfer (FRET) assay

Granulocytes were fixed with 2 % formaldehyde for 30 min, washed with 0.1 % glycine, and permeabilized with 0.01 % saponin for 15 min. The cells were incubated for 2 h with goat anti-P2Y_1_ receptor (4 μg/ml, Santa Cruz). Goat anti-IgG-Alexa Fluor 488 conjugated antibody (Invitrogen, USA) was used for 40 min as secondary antibody. Subsequently, rabbit anti-P2Y_2_ or rabbit anti-P2Y_4_ were used (Alomone, Israel). Rabbit anti-IgG-Alexa Fluor 546 conjugated antibody (Invitrogen, USA) was used for 40 min. Nuclei were stained with DAPI (20 μg/ml, Sigma, USA) for 20 min. Light microscopy analyses were performed with a confocal laser scanning microscope equipped with a Plan-Apochromat 63x objective (Leica, SP8). The pinhole device was adjusted to capture fluorescence of one Airy unit in one focal section.

For FRET analysis, FRET signal was obtained at 488 nm excitation employing an argon laser and the emitted fluorescence was detected from 560 nm to 650 nm in 10.6 nm steps using the lambda modus. FRET ratios were calculated using the following equation: FRET ratio = Intensity at 575 nm/Intensity at 521 nm [20].

In addition, emission spectra were measured at 488 nm excitation and the emission fluorescence was detected from 515 nm to 600 nm in 1 nm steps using FLEX station 3 (Molecular devices, USA). Fluorescence intensities were normalized to the emission maximum of the P2Y_1_ receptor (donor) at 525 nm. FRET ratios were calculated using the following equation: FRET ratio = Intensity at 575 nm/Intensity at 525 nm.

### Purinergic quantification

Quantification of ATP and its analogs was carried out by Liquid Chromatography-Electrospray Ionization Mass Spectrometry (LC/ESI-MS) analysis as described previously [21, 22]. Calibration samples of ATP and analogs, at 10^−3^ M concentration, were prepared in a water:methanol mixture (50:50 v/v). LC/ESI-MS data were obtained on a Waters instrument, model 3100 coupled on a Waters Alliance model 2695 system and a Waters detector model 2847, using a Waters Nova-Pak C_18_ column (2.1 × 150 mm, 60 Å, 3.5 μm); solvent A: aqueous DEA (0.01 M) and solvent B: aqueous ammonium acetate (0.1 M); 40 % B isocratic elution for 15 min, λ = 254 nm. Mass measurements were performed in a negative mode in the following conditions: mass range between 100 and 1000 m/z; nitrogen gas flow: 4.1 L/h; capillary: 3.9 kV; cone voltage: 47 V; extractor: 8 V; source heater: 105 °C; solvent heater: 400 °C; ion energy: 1.0 V and multiplier: 996 V.

### Statistical analysis

In cell suspensions, cells were loaded with fura-2 fluorophore. The cytosolic Ca^2+^ values were expressed as [Ca^2+^]_cyt_. In cells loaded with fluo-4 fluorophore, [Ca^2+^]_cyt_ values are shown as a representative pseudocolored image with reference to a fluorescence intensity scale [0 = black, 255 = white], and the fluorescence intensity is normalized by basal intensity.

Data are expressed as the mean ± standard error of the mean (SEM). Statistical comparisons were performed using Student’s T-test or analysis of variance (ANOVA), with the Dunnett’s post hoc test. Values of *P* < 0.05 were considered statistically significant. All graphical data represent at least three independent experiments.

## Results

### Presence of P2Y receptors in granulocytes

**Fig. 1:**
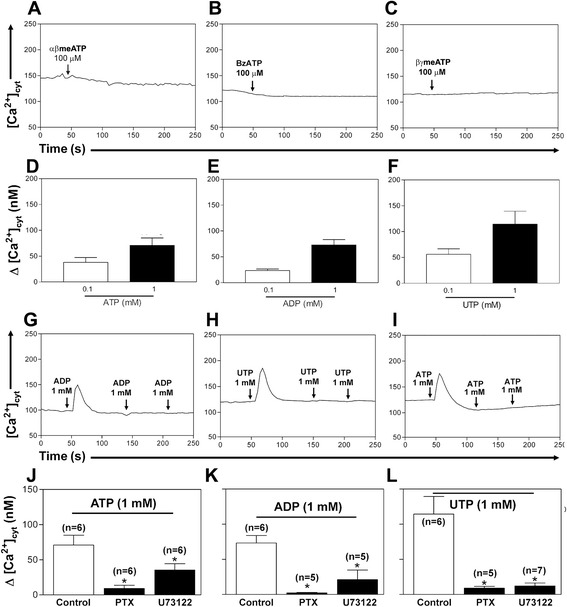
Characterization of P2 responses in mice granulocytes. Granulocytes were loaded with the Ca^2+^ indicator fura-2. The P2X receptors agonists **a** αβMeATP, **b** BzATP and **c** βγMeATP did not induce increases in [Ca^2+^]_cyt_. **d**–**f** Average increase in [Ca^2+^]_cyt_ response to these agonists. However **g** ATP, **h** ADP and **i** UTP were able to induce a transient increase in [Ca^2+^]_cyt_; one application of a high agonist concentration promoted desensitization of the ATP response. **j**–**l** Granulocytes were pretreated for 3 h with PTX (0.05 μg/ml) or U73122 (5 μM). ATP and its analogs showed the same sensitivity to G_i_-coupled protein inhibitor PTX and PLC inhibitor U73122. These results are the means ± SEM of [Ca^2+^]_cyt_ increase above the resting level. *P < 0.05, ANOVA test

Since most activated P2 receptors cause a [Ca^2+^]_cyt_ uptake, this calcium was monitored in granulocytes to determine the P2 receptor subtypes expressed by these cells. P2X agonists BzATP (10–100 μM), αβMeATP (10–100 μM) and βγMeATP (1–10 μM) did not raise the [Ca^2+^]_cyt_ levels (Fig. 1a–c). High concentrations of ATP, ADP and UTP (~100 μM to 1 mM) were required to promote maximal increases in [Ca^2+^]_cyt_ in granulocytes (Fig. 1d–f), as previously reported for some hematopoietic cell types [5, 17, 18, 22–24]. When granulocytes were stimulated with a single application, concentrations of ATP, ADP and UTP around 1 mM produced maximal increases in [Ca^2+^]_cyt_ (Fig. 1d–f). Subsequent treatment with ATP (1 mM) and analogs initially induced a transient increase in [Ca^2+^]_cyt_ and homologous desensitization (Fig. 1g–i).

ADP and UTP are typical P2Y agonists. To confirm that the Ca^2+^ release is due the activation of P2Y receptors, Pertusis toxin (PTX), a G_i_ protein-coupled receptor inhibitor, and U73122, a PLC inhibitor were used. PTX and U73122 caused a decrease in the agonist-dependent increases of [Ca^2+^]_cyt_ (Fig. 1j–l).

**Fig. 2:**
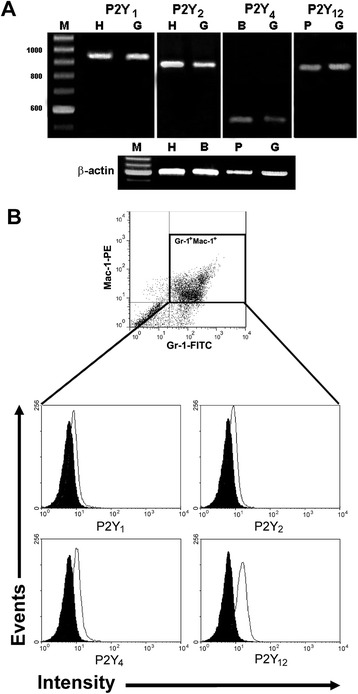
Presence of P2Y receptors in bone marrow granulocytes. **a** Granulocytes expressed mRNA for P2Y_1_, P2Y_2_, P2Y_4_ and P2Y_12_ receptors. Ladder 100-bp marker [M]; heart [H]; brain [B]; platelets [P]; granulocytes [G]. **b** To analyze the expression at the protein level of P2Y receptors in Gr-1^+^Mac-1^+^ population (dot plot) flow cytometry was used. Solid black histogram: negative control (preincubated with the peptide antigen); open histogram: cells labeled with the respective antibodies

The existence of P2Y receptors was confirmed by RT-PCR and flow cytometry. RT-PCR assays showed expression of mRNA for ADP and UTP receptors: P2Y_1_, P2Y_2_, P2Y_4_ and P2Y_12_ (Fig. 2a). Flow cytometry confirmed the presence of P2Y_1_, P2Y_2_, P2Y_4_ and P2Y_12_ receptors in granulocytes (Gr-1^+^Mac-1^+^ cells) of bone marrow at the protein level (Fig. 2b). These are receptors activated by ADP (P2Y_1_ and P2Y_12_) and UTP (P2Y_2_ and P2Y_4_).

### Heteromeric association of P2Y_1_ receptor with P2Y_2_ and P2Y_4_ receptors in granulocytes

**Fig. 3:**
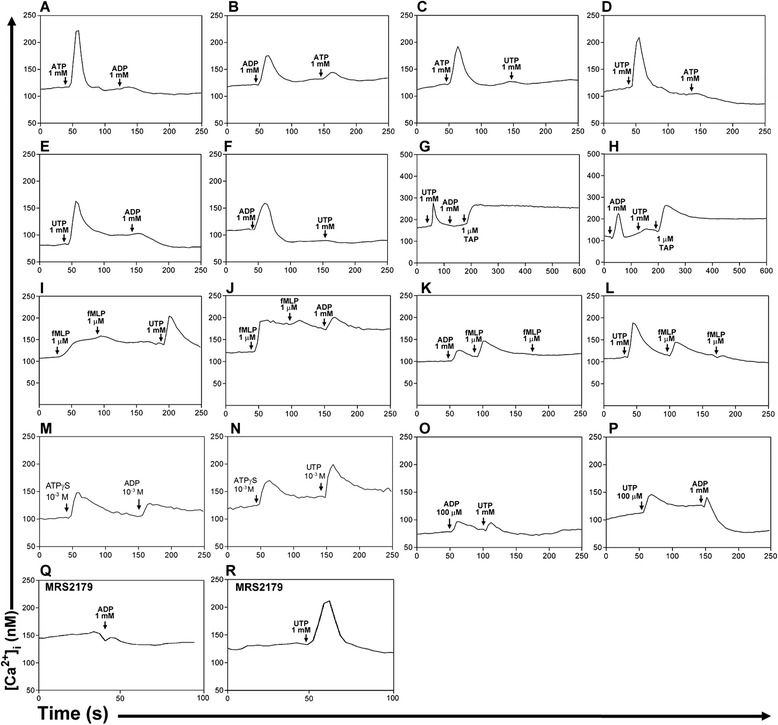
Cross-desensitization among ATP, ADP and UTP in mice granulocytes. Granulocytes were loaded with the Ca^2+^ indicator fura-2. **a**–**d** Addition of ATP fully inhibited responses to ADP and UTP and vice-versa. **e**, **f** ADP and UTP showed full heterologous desensitization. **g**, **h** Thapsigargin, a Ca^2+^-ATPase inhibitor, which releases Ca^2+^ from intracellular stores, showed that the lack of a second response was not due to emptying of intracellular Ca^2+^ stores. **i**–**l** fMLP, another G-protein-coupled agonist, showed the specificity of cross-desensitization between ADP and UTP. **m**, **n** ATPγS did not promote heterologous desensitization to ADP and UTP response. **o**, **p** Lower concentration of ADP and UTP reduced responses to ADP and UTP. **q**, **r** MRS2179, a specific antagonist of the P2Y_1_ receptor, inhibited responses to ADP but not to UTP. Granulocytes were pretreated for 30 min with MRS2179. All data are representative responses of at least four independent experiments

Initially, cross-desensitization among ATP, ADP and UTP was used to investigate the presence of distinct P2 receptors in granulocytes. As expected, ATP abolished ADP and UTP responses (Fig. 3a, c). However, ADP and UTP also completely abolished ATP responses (Fig. 3b, d). Moreover, ADP and UTP showed full heterologous desensitization (Fig. 3e, f). These results suggested that ADP and UTP act in the same P2Y receptor, though this found was not observed in any other P2 receptors in mammals.

Additionally, thapsigargin, a Ca^2+^-ATPase inhibitor, avoided Ca^2+^ re-uptake into endoplasmatic reticulum, showing that the lack of a second response to ADP and UTP was not due to a deployment of intracellular Ca^2+^ stores or a technique artifact (Fig. 3g, h). To confirm that the cross-desensitization between ADP and UTP was a specific response, N-formyl-methionine-leucine-phenylalanine (fMLP), another granulocyte-activation related G-protein-coupled agonist, was used. No cross-desensitization between fMLP with ADP or UTP was observed (Fig. 3i–l). In addition, ATPγS, an analogue of ATP that does not activate P2Y_1_ or P2Y_2_/P2Y_4_ receptors, did not promote cross-desensitization (Fig. 3m, n). ADP and UTP (Fig. 3o–p). To evaluate the participation of P2Y_1_ receptor, its specific antagonist MRS2179 was used. MRS2179 was able to abolish the ADP response (Fig. 3q), but not the UTP response (Fig. 3r).

None of the P2 receptors are activated by both ADP and UTP in mammals, except the P2y_3_ receptor which was cloned from a chick brain [25] and was activated by one as well as another. Another possibility is that the presence of a new P2Y receptor or association of P2Y_1_ receptor with other UTP receptors, such as P2Y_2_ or P2Y_4_ receptors, could change their pharmacological characteristics, since homo- and hetero-oligomerization is common for G protein-coupled receptors [7].

**Fig. 4:**
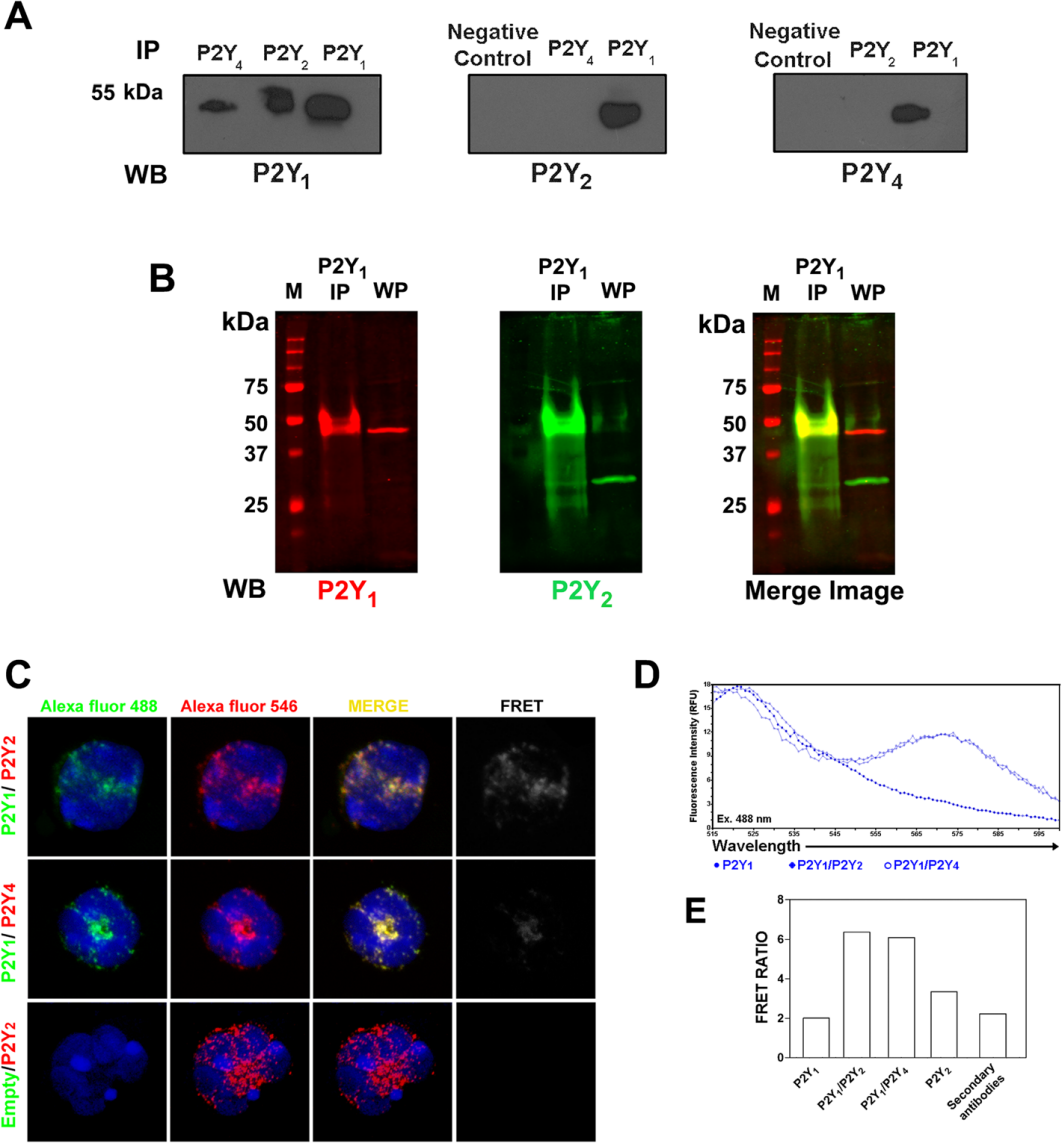
Heteromeric association among P2Y receptors. **a** Immunoprecipitation (IP) of cell lysates visualized by western blot (WB) analyses. **a** Immunoprecipitated receptors P2Y_2_ and P2Y_4_ were visualized with an anti-P2Y1 antibody. P2Y_1_ receptors immunoprecipitated by the same antibody were used as positive controls. Additionally, immunoprecipitated receptor P2Y_1_ was seen with either anti-P2Y_2_ or anti-P2Y_4_ antibodies. Unspecific immunoprecipitates of rabbit normal serum were used as negative controls (NC). **b** Immunopreciptation with infrared fluorescent secondary antibody: P2Y_1_ receptors were immunopreciptated with goat antibodies. After SDS-PAGE the transferred proteins were revealed with rabbit antibodies and analyzed with donkey anti-rabbit antibodies to different fluorophores. First for P2Y_1_ (red), and after membrane stripping for P2Y_2_ (green). The whole protein lysate (WP) was used as a control. **c** Confocal images show the colocalization of P2Y_1_ receptor with P2Y_2_ or P2Y_4_ receptor. FRET signal evidence that these receptors are spatially close. **d** To corroborate confocal images, emission spectra of samples are shown (Ex. 488). **e** Analysis of FRET ratios were calculated as explained in the Methods section

To verify whether P2Y_1_ receptors were associated with other P2Y receptors, a coimmunoprecipitation assay was performed involving P2Y_1_, P2Y_2_ and P2Y_4_ receptors. P2Y_1_ receptor was immunoprecipitated with P2Y_2_ and P2Y_4_ antibodies; immunoprecipitation of P2Y_2_ and P2Y_4_ receptors were also reveled with P2Y_1_ antibody (Fig. 4a). Additionally, an immunoprecipitation assay was performed using fluorescence probes to confirm the presence of both receptors in the same band; P2Y_1_ and P2Y_2_ receptor are present together in the same band with a molecular weight around 50 kDa (Fig. 4b). To corroborate this hypothesis of the association of P2Y receptors a FRET assay was employed. Confocal microscopy showed P2Y_1_/P2Y_2_ and P2Y_1_/P2Y_4_ receptors colocalization (Fig. 4c Merge), and FRET signal was obtained when the sample was excited at 488 nm with the argon laser (Donor: Alexa Fluor 488) and emission wavelength was captured at 560–590 nm (Acceptor: Alexa Fluor 546 - emission of P2Y_2_ or P2Y_4_ receptor) (Fig. 4c). Moreover, emission spectra of the samples were measured in a microplate spectrofluorometer. As observed in Fig. 4d the sample labeled with antibodies P2Y_1_/P2Y_2_ and P2Y_1_/P2Y_4_ showed a second peak at 575 nm of FRET signal. Quantification of FRET signal is shown in Fig. 4e. Therefore, heteromeric association between P2Y_1_ and P2Y_2_ or P2Y_4_ receptors also occurred in the granulocytes. This could explain the changes observed in the classical pharmacology of P2Y receptors in mice granulocytes, where P2Y receptors were simultaneously activated by ADP/UTP, but with low affinity.

ATP and analogs are unstable compounds and as high concentrations were used to stimulate granulocytes, it was decided to quantify the presence of degraded products in ATP, ADP and UTP by using liquid chromatography coupled to mass spectrometry. Analytical evaluation detected that the ATP sample (78 %) contains ADP (9.6 %) and adenosine (3.5 %); ADP sample (88 %) contains AMP (8.5 %) and adenosine (3.5 %); and UTP sample (40 %) contains UDP (49 %) and UMP (11 %). However, neither contamination of UTP was detected in ADP sample and ADP was neither detected in the UTP sample.

### Heteromeric association may occur in mice myelocytic progenitors

**Fig. 5:**
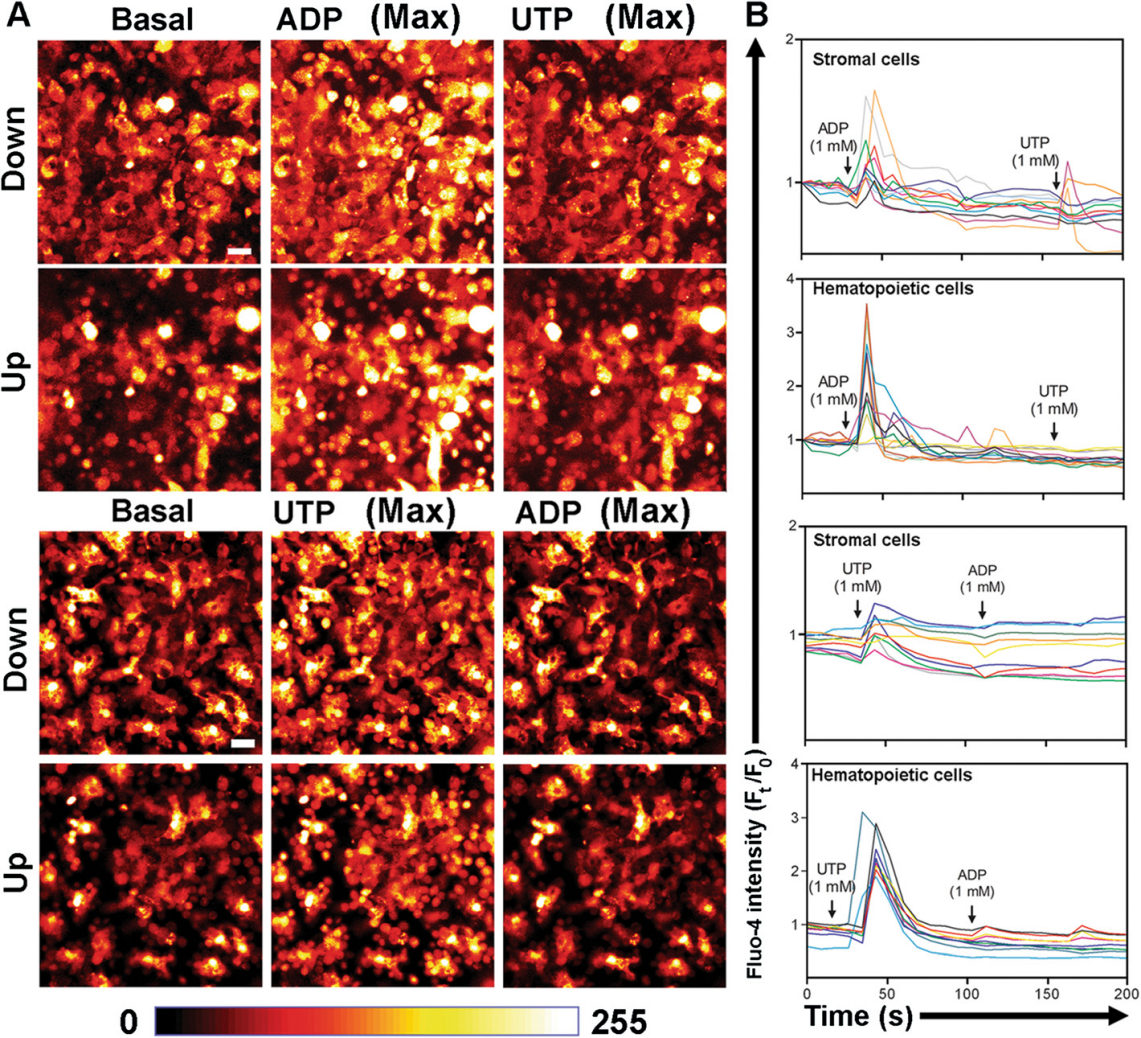
Heterologous desensitization between ADP and UTP also occurs in hematopoietic progenitor cells. Cells were loaded with the Ca^2+^ indicator fluo-4, and intensity was measured with confocal microscopy (LSM 510 META). Scale bar = 20 μM. The images are a pseudocolored representation in reference to the fluorescence intensity scale (0 = black, 255 = white). **a** Images in two Z planes showed cell preparations before agonist addition (basal) and after stimulation (max) with 1 mM of ADP and UTP. **b** Records of fluorescence intensity as a function of time corresponded to images of hematopoietic and stromal cells in **a**, and represented responses of four independent experiments

The effects of ADP and UTP on [Ca^2+^]_cyt_ increase in long-term bone marrow cultures containing myelocytic progenitors were also analyzed. In these cultures, all hematopoietic cells (smaller cells with circular shape) were responsive to ATP, ADP and UTP showing the same heterologous response observed in granulocytes (Fig. 5). Heterologous desensitization was also observed in many stromal cells (large cells with variable shapes) of hematopoietic origin.

## Discussion

The response exhibited by mice bone marrow granulocytes during characterization of P2 receptors is unique among mammalian P2 receptors. The avian P2y3 receptor [25], ortholog of the mammalian P2Y_6_ receptor [26], is the only known receptor activated by ADP and UTP.

The presence of a new receptor with distinct pharmacological characteristics in granulocytes seems to be unlikely. Therefore, we have presented another possibility: the presence of direct or indirect heteromeric association among P2Y receptors. Among P2Y receptors, ADP- (P2Y_1_) and UTP-activated (P2Y_2_ and P2Y_4_) receptors were the most likely candidates. As observed in Fig. 4, coimmunoprecipitation and FRET assays revealed heteromeric association of P2Y_1_-P2Y_2_ and P2Y_1_-P2Y_4_ receptors. However, no association between P2Y_2_ and P2Y_4_ receptors was observed (Fig. 4a). Heteromeric associations involving P2Y_1_-P2Y_2_ and P2Y_1_-P2Y_4_ receptors could account for the unexpected pharmacology characteristic observed in P2Y receptors in granulocytes.

Heterodimerization involving P2X receptors is already known; the receptor heterodimers P2X_2–3_, P2X_2–6_, P2X_4–6_ and P2X_1–5_ have been characterized extensively. Heteromeric association between P2Y receptors has also been observed in transfected systems, but it has never been reported that this phenomenon occurs in primary cells. Yoshioka et al. [3] observed heteromeric association involving P2Y_1_ and A_1_ receptors after receptor transfection of human embryonic kidney (HEK293T) lineage cells. Ecke et al. [4], also working with transfected HEK293 cells, described the first hetero-oligomerization among P2Y (P2Y_1_ and P2Y_11_) receptors. Additionally, homodimeric and oligomer complexes formed by P2Y_2_, P2Y_4_ and P2Y_12_ receptors may be typical for these receptors [5–7]. Among P2Y receptors, P2Y_4_ and P2Y_6_ receptors have a secondary structure that makes them likely to associate with other P2Y receptors [7]. The present results support the hypothesis that heteromeric association among P2Y receptors is common in bone marrow granulocytes.

Purinergic agonist affinity was low in granulocytes. In some hematopoietic lineages (e.g., erythroid and myeloid lineages), purinergic agonist affinity for P2Y receptor has been found to be lower than in other cell types [17, 18, 22–24, 27]. Homo- and hetero-association between receptors could also have changed G-protein selectivity and agonist efficiency.

The fast degradation of ATP and analogs may be related to the low activity observed by these agonists. However, as no traces of UTP were detected in the sample of ADP, or ADP in the sample of UTP, desensitization by contamination was excluded.

Usually, P2Y_1_, P2Y_2_, P2Y_4_ and P2Y_6_ receptors are associated with G_q/11_ proteins, whereas P2Y_11_, P2Y_12_, P2Y_13_ and P2Y_14_ receptors are associated with G_i_ proteins. Murthy & Makhlouf [26] showed the complexity of P2Y receptor signaling in smooth muscle cells, where activation of P2Y_2_ receptors triggered both PLCβ1 via G_α11_ and PLCβ3 via G_βγi3_. The occurrence of homo- and hetero-association involving P2Y receptors would create more opportunities for intracellular pathways associated with these receptors. It is also necessary to account for raft microdomains on the cellular membrane. Together with the cytoplasm, these microdomains can create distinct intracellular pathways, thus explaining how similar intracellular signaling systems can trigger different effects. This is supported by reports showing the presence of P2Y homo-oligomers and homo-dimers in lipid raft fractions [6, 7].

The presence of P2Y receptors in hematopoietic cells supports the assertion that these receptors are involved in proliferation and differentiation. The effect of ATP has been studied in human myelocytic lineage HL-60, which expresses P2Y_2_ receptors. Differentiation of HL-60 cells to granulocytes did not alter P2Y_2_ receptor expression. However, differentiation into monocytes/macrophages decreased P2Y_2_ receptor expression [28]. Adrian et al. [29] showed alteration of expression of P2 receptors during granulocytic and monocytic differentiation of HL-60 cells. Heterologous desensitization with ADP and UTP was also observed on cobblestone areas in long-term bone marrow cultures (Fig. 5), suggesting that hetero-association may be present in primitive cells. Recently, the participation of P2 receptors in differentiation into myelocytes has been confirmed in hematopoietic stem cells [17, 22, 24]. Hetero-association among P2Y receptors would be a characteristic of the myelocytic lineage in mice.

## Conclusion

The results of the present study demonstrate that granulocytes express P2Y_1_, P2Y_2_ and P2Y_4_ receptor, and heteromeric association between P2Y_1_/P2Y_2_ and P2Y_1_/P2Y_4_ occur.
